# Digestibility of Oysters

**Published:** 1883-01

**Authors:** 


### THE DIGESTIBILITY OF OYSTERS.

Why oysters should be eaten raw is explained by Dr. William Roberts in his lecture on "Digestion." He says that the general practice of eating the oyster raw is evidence that the popular judgment upon matters of diet is usually trustworthy. The fawn-colored mass, which is the delicious portion of the fish, is its liver, and is simply a mass of glycogen. Associated with the glycogen, but withheld from actual contact with it during life, is its appropriate digestive ferment—the hepatic diastase. The mere crushing of the oyster between the teeth brings these two bodies together, and the glycogen is at once digested without any other help than the diastase. The raw, or merely warmed oyster, is self-digestive. But the advantage of this provision is wholly lost by cooking; for the heat immediately destroys the associated ferment, and a cooked oyster has to be digested, like any other food, by the eater's own digestive powers.

"My dear sir, do you want to ruin your digestion?" asked Professor Houghton of Trinity College one day of a friend who had ordered brandy and water with his oysters in a Dublin restaurant.

Then he sent for a glass of brandy and a glass of Guinness's XX, and put an oyster in each. In a very short time there lay in the bottom of the glass of brandy a tough, leathery substance resembling the finger of a kid glove, while in the porter there was hardly a trace of the oyster to be found.

